# Hazardous drinking by age at migration and duration of residence among migrants in Sweden

**DOI:** 10.1111/dar.13982

**Published:** 2024-11-25

**Authors:** Lisa Berg, Sol P. Juárez, Helena Honkaniemi, Mikael Rostila, Andrea Dunlavy

**Affiliations:** ^1^ Department of Public Health Sciences Stockholm University Stockholm Sweden; ^2^ Centre for Health Equity Studies Stockholm University/Karolinska Institutet Stockholm Sweden; ^3^ Aging Research Center Karolinska Institutet/Stockholm University Stockholm Sweden

**Keywords:** age at arrival, alcohol drinking, gender differences, immigrants, length of stay

## Abstract

**Introduction:**

Sweden, with its history of restrictive alcohol policies and a large and diverse migrant population, constitutes an interesting context for studies on alcohol consumption patterns in migrant groups. This study examines how hazardous drinking among migrants in Sweden varies by origin, duration of residence and age at migration.

**Methods:**

Pooled cross‐sectional survey data from the Västra Götaland region of Sweden, collected in 2011 and 2015, were linked to register data containing demographic, socioeconomic and migration‐related factors (i.e., country of birth, duration of residence, age at migration), for 7754 migrants and 68,493 Swedish‐born individuals aged 18–84 years. Logistic regression analyses were applied to estimate odds ratios (OR) and 95% confidence intervals (CI) for hazardous drinking, identified by the validated Alcohol Use Disorders Identification Test.

**Results:**

Relative to Swedish‐born individuals, migrants from other Nordic countries had higher odds of hazardous drinking (OR 1.45, 95% CI 1.18–1.77), while migrants from other European (OR 0.55, 95% CI 0.44–0.69) and non‐European (OR 0.25, 95% CI 0.20–0.31) countries showed lower likelihoods. Among non‐Nordic migrants in particular, hazardous drinking was more common among those with a longer duration of residence and those who migrated at pre‐school ages.

**Discussion and Conclusions:**

Hazardous drinking among non‐Nordic migrants increasingly resembled that of the Swedish‐born population over time, aligning with findings in less restrictive alcohol policy contexts. Understanding how drinking patterns vary among migrant groups over time and across policy contexts is essential for developing effective public health strategies to reduce hazardous consumption and associated health and social harms.

**Key Points**

- Register‐linked survey data was used to study hazardous alcohol consumption in a large migrant population using a validated screening tool.
- The Swedish setting, with restrictive alcohol policies aimed at maintaining low overall consumption, offers a particularly interesting context for studies on alcohol consumption patterns in migrant groups.
- Patterns of alcohol consumption among migrants differed by origin, duration of residence and age at migration, with hazardous drinking being more common among those with a longer duration of residence and those who migrated at pre‐school ages.
- Non‐Nordic migrants in Sweden displayed patterns of convergence to native levels of hazardous drinking with increased exposure to the settlement context, results that align with findings from countries with less restrictive alcohol policy approaches.

## 1 INTRODUCTION

Harmful use of alcohol is a leading risk factor for poor health and premature mortality worldwide [1]. Among migrant populations, health risk behaviours such as alcohol consumption are influenced by a complex interplay of factors related to the country of origin, the migration experience, the settlement context and integration processes. Relative to native‐born populations, foreign‐born individuals (hereafter migrants), especially recent arrivals, often exhibit lower levels of alcohol consumption [2]. However, as duration of residence in the new country increases, a convergence to native consumption levels has been observed. The majority of existing evidence on consumption patterns among migrant groups stems from North American contexts, while European studies, particularly from the Nordic countries, are scarce and the findings more conflicting [2]. Previous research also suggests an important role of the timing of migration in the life course in shaping consumption patterns, with individuals arriving at younger ages often displaying higher levels of consumption and elevated risks of hazardous drinking later in life, relative to those who arrive at later ages [3]. Changes in alcohol consumption habits, along with other health risk behaviours, have been proposed to partially explain the general health decline frequently observed over time among migrants in the settlement country [4].

Levels of alcohol consumption and hazardous drinking, which refers to drinking patterns that reflect an increase in risks of acute or chronic negative alcohol‐related outcomes [5], vary between and within countries [6]. Nordic studies have shown less frequent alcohol use [7, 8] and binge drinking [8] among migrant populations relative to the native‐born majority. Heavy and binge drinking has been associated with younger age at arrival in Finland and Sweden [8, 9], whereas longer residence has been associated with less frequent drinking in Norway [7]. A handful of Nordic register‐based studies have explored the risk of alcohol use disorders (AUD) among migrants based on hospitalisation data, reflecting chronic, regular alcohol use and the more severe manifestation of alcohol misuse based on clinical diagnosis, rather than overall consumption patterns [5]. These studies generally demonstrate a lower risk of AUD among non‐Nordic migrants [10, 11, 12, 13, 14] but increased rates of AUD with earlier age at migration [12] and longer residence [11].

Sweden offers a particularly interesting context to study alcohol consumption patterns in the population. It has implemented restrictive alcohol policies and maintains state‐controlled alcohol retail monopolies, in order to facilitate the prioritisation of welfare and the mitigation of alcohol‐related harms over profit motives, thus favouring a public health perspective [15]. Sweden also stands out with its large migrant population and long history of migration; in recent decades, Sweden has experienced a substantial increase in migration flows, with approximately 20% of the current population being foreign‐born [16]. Beginning in the late 1940s and continuing through the 1960s, migration to Sweden was primarily labour‐driven, with individuals arriving from other Nordic countries as well as southern and central Europe. In subsequent decades, migration shifted towards refugees and asylum seekers fleeing wars and conflicts in countries outside of Europe. During the 1970s and 1980s, migrants came mainly from Latin America, East Africa and Iran, and in the 1990s, migration primarily included individuals from the former Yugoslavia and Soviet Union, Somalia and Iraq [17]. Migration increased into the 2000s, peaked in 2016, and then declined, largely due to a decrease in asylum immigration. Gaining a deeper understanding of consumption patterns within this diverse and growing foreign‐origin population is of great public health relevance.

The aim of the current study was to examine hazardous drinking among migrant men and women in Sweden using population‐representative, register‐linked survey data and a validated screening instrument. Our analysis specifically focuses on how consumption patterns vary by region of origin, duration of residence and age at migration.

## 2 METHODS

### 2.1 Data material and study population

Data were obtained from the population‐representative cross‐sectional *Health on Equal Terms* surveys conducted in the Swedish county of Västra Götaland in 2011 and 2015, administered by the Public Health Agency of Sweden together with Statistics Sweden [18, 19]. Data consisted of information from self‐administrated questionnaires and linked register data for a random selection of individuals aged 16 to 84 years. The response rate was 54.5% in 2011 and 55.5% in 2015, with lower survey response rates among European (2011: 42%; 2015: 39%) and non‐European (2011: 32%; 2015: 28%) migrants [18, 19]. The study population consisted of pooled data for 68,493 Swedish‐born and 7754 foreign‐born individuals aged 18–84 years at the time of the survey, who participated in either 2011 or 2015, after exclusion of individuals with missing information on any of the study variables (i.e., missing on the three AUDIT‐C questions [0.5%], education [0.3%], income [0.2%] or psychological distress [2.7%]).

All participants provided informed consent for linkage of register data and the use of collected data for research purposes. Ethical approval for the research was obtained from the Regional Ethical Review Board in Stockholm, 2017/716‐31/5.

### 2.2 Hazardous drinking

The Alcohol Use Disorders Identification Test (AUDIT), developed by the World Health Organization, is an established screening tool designed to identify hazardous and harmful drinking, defined as patterns of alcohol use that increase the risk of future alcohol problems and chronic and acute alcohol‐related harms [20]. The Health on Equal Terms surveys included AUDIT‐C, a modified three‐question version of AUDIT that has been validated and extensively used in epidemiological surveys [21]. The three questions cover frequency and amount of drinking and the frequency of binge drinking: (i) How often did you have a drink containing alcohol during the past 12 months? (response options: ‘4 times a week or more’, ‘2‐3 times a week’, ‘2‐4 times a month’, ‘1 time a month or more rarely’ and ‘Never’); (ii) How many drinks did you have on a typical day when you were drinking during the past 12 months? (response options: ‘1–2’, ‘3–4’, ‘5–6’, ‘7–9’ and ‘10 or more’); and (iii) How often did you have six or more drinks on one occasion during the past 12 months? (response options: ‘Daily or almost daily’, ‘Every week’, ‘Every month’, ‘More rarely than once a month’ and ‘Never’). Each response was scored 0–4 points (with higher points indicating greater frequency and amount), summed up to a maximum score of 12 points, and dichotomised. Cut‐offs to indicate hazardous drinking were set to a score of 6 or more for men, and 5 or more for women, in line with standards set by the Public Health Agency of Sweden [22]. Individuals with a score of zero were defined as abstainers.

### 2.3 Migration background characteristics

Register‐information on the following categories of country/region of birth was available: Africa, Asia, Europe (excluding the Nordic countries), Nordic countries (excluding Sweden), North America, Oceania, Sweden, South America and ‘Others’. Based on previous research, we expected Nordic migrants to have higher risks of hazardous use [10, 23], and for there to be differences in hazardous use between European and non‐European migrants [8, 11, 24]. Based on these previously used categorisations, and to ensure statistical power for our analyses, country/region of birth was grouped as ‘Swedish‐born’, ‘Nordic countries’, ‘European countries’ and ‘non‐European countries’.

Duration of residence at the time of the survey, that is, years since residency in Sweden was established, was categorised as <10, 10–19 and ≥20 years. Age at migration was categorised as <7, 7–17, 18–30 and >30 years. This categorisation was chosen to reflect life stages of potential importance (pre‐school age, school age, young adulthood, adult life) in relation to hazardous drinking in adulthood.

### 2.4 Covariates

Register information on demographic (age at the year of the survey and gender) and socio‐economic (education and income) factors was also linked to the survey data. The existence of gender differences in alcohol consumption are well documented [25]. Moreover, a growing body of evidence indicates that the migration process and its ramifications for health behaviours and health outcomes differ by gender, underscoring the need to study synergistic effects at the intersections of migration and gender [26]. Educational level and income were included as covariates, as hazardous consumption tends to be socially patterned [24, 27]. Educational level was categorised as low (up to 2 years of secondary education), medium (up to 2 years of university/college) or high (graduate and postgraduate studies). Individual disposable income was divided into quintiles. Hazardous drinking is also associated with mental health [28], with poor mental health being more common among migrant populations compared with natives [29]. Psychological distress was measured using the validated 12‐item version of the General Health Questionnaire [30].

### 2.5 Statistical analysis

Logistic regression analyses were used to estimate odds ratios (OR) and 95% confidence intervals (CI) for the association between country/region of birth, duration of residence and age at migration and hazardous drinking separately. Results are presented for a first model including gender, age at survey (included as a continuous variable) and survey year (Model 1), and a second model adding socioeconomic variables (educational level and income) and psychological distress (Model 2). To account for non‐response and potential socio‐demographic differences between respondents and non‐respondents, survey weights provided by Statistics Sweden were applied. Information from the Total population register and the Education register on age, gender, education, region of birth, marital status, and living in urban area was used to create the survey weights [18, 19]. Post‐estimation tests were run to assess for statistically significant differences in OR estimates for hazardous drinking by duration of residence and age at migration in the general migrant population and within each migrant category.

To gain a deeper understanding of how patterns of hazardous drinking differ among men and women in relation to different migration background characteristics, all analyses were stratified by gender and interaction analyses between gender and migration background characteristics were performed.

All analyses were performed using Stata Statistical Software, release 15.

## 3 RESULTS

Table 1 presents descriptive statistics summarising the characteristics of the study population. The migrant population (10.2% of the study population) consisted of 2576 individuals born in other Nordic countries, 2834 individuals born in other European countries and 2344 from non‐European countries (Asia 69.3%, Africa 12.8%, South America 10.1%, North America 5.6%, Oceania 0.9% and other unspecified countries 1.3%). The distribution of AUDIT‐C scores differed substantially by migrant origin (Table S1, Supporting Information). The proportion of abstainers was highest among individuals with non‐European origin (41.1%) and lowest among individuals born in Sweden (13.2%). The highest proportions of hazardous drinking were seen among individuals born in Sweden or other Nordic countries, and the lowest among European and non‐European women and non‐European men.

**TABLE 1:** Descriptive characteristics by country/region of birth and gender.

	Sweden	All migrants	Migrant women, by country of origin			Migrant men, by country of origin		
			Other Nordic	European	Non‐European	Other Nordic	European	Non‐European
	*n* = 68,493	*n* = 7754	*n* = 1453	*n* = 1562	*n* = 1234	*n* = 1123	*n* = 1272	*n* = 1110
Age at survey, mean (SD)	53.5 (17.9)	52.5 (16.7)	61.3 (13.4)	50.9 (17.0)	42.8 (13.9)	62.4 (13.1)	51.9 (17.0)	45.0 (15.2)
Duration of residence								
≥20 years	–	4392 (56.6)	1127 (77.6)	802 (51.3)	499 (40.4)	800 (71.2)	635 (49.9)	529 (47.7)
10–19 years	–	1541 (19.9)	142 (9.8)	408 (26.1)	309 (25.0)	138 (12.3)	331 (26.0)	213 (19.2)
<10 years	–	1821 (23.5)	184 (12.7)	352 (22.5)	426 (34.5)	185 (16.5)	306 (24.1)	368 (33.2)
Age at migration								
<7 years	–	838 (10.8)	171 (11.8)	143 (9.2)	167 (13.5)	150 (13.4)	99 (7.8)	108 (9.7)
7–17 years	–	1050 (13.5)	242 (16.7)	196 (12.6)	133 (10.8)	156 (13.9)	167 (13.1)	156 (14.1)
18–30 years	–	3206 (41.4)	680 (46.8)	678 (43.4)	483 (39.1)	426 (37.9)	520 (40.9)	419 (37.8)
>30 years	–	2660 (34.3)	360 (24.8)	545 (34.9)	451 (36.6)	391 (34.8)	486 (38.2)	427 (38.5)
Educational level								
Low	34,910 (51.0)	4005 (51.7)	955 (65.7)	644 (41.2)	525 (42.5)	809 (72.0)	561 (44.1)	511 (46.0)
Middle	21,887 (32.0)	2179 (28.1)	284 (19.6)	502 (32.1)	386 (31.3)	225 (20.0)	445 (35.0)	337 (30.4)
High	11,696 (17.1)	1570 (20.3)	214 (14.7)	416 (26.6)	323 (26.2)	89 (7.9)	266 (20.9)	262 (23.6)
Income								
Quintile 1 (lowest)	12,816 (18.7)	2488 (32.1)	424 (29.2)	516 (33.0)	509 (41.3)	325 (28.9)	330 (25.9)	384 (34.6)
Quintile 2	13,875 (20.3)	1715 (22.1)	391 (26.9)	358 (22.9)	257 (20.8)	244 (21.7)	250 (19.7)	215 (19.4)
Quintile 3	14,138 (20.6)	1412 (18.2)	284 (19.6)	321 (20.6)	240 (19.5)	177 (15.8)	215 (16.9)	175 (15.8)
Quintile 4	14,021 (20.5)	1222 (15.8)	216 (14.9)	230 (14.7)	155 (12.6)	190 (16.9)	244 (19.2)	187 (16.9)
Quintile 5 (highest)	13,643 (19.9)	917 (11.8)	138 (9.5)	137 (8.8)	73 (5.9)	187 (16.7)	233 (18.3)	149 (13.4)
Psychological distress, yes	9327 (13.6)	1553 (20.0)	260 (17.9)	382 (24.5)	312 (25.3)	145 (12.9)	192 (15.1)	262 (23.6)
Hazardous drinking, yes	9540 (13.9)	746 (9.6)	171 (11.8)	85 (5.4)	58 (4.7)	197 (17.5)	145 (11.4)	90 (8.1)

*Note*: Numbers are *n* (%) unless otherwise indicated.

ORs for hazardous drinking by migration characteristics are presented in Table 2. Relative to Swedish‐born individuals, migrants from Nordic countries were more likely to report hazardous drinking (OR 1.45, 95% CI 1.18–1.77) after adjustment for socio‐demographic variables and psychological distress. Migrants from European (OR 0.55, 95% CI 0.44–0.69) and non‐European (OR 0.25, 95% CI 0.20–0.31) countries were less likely than Swedish‐born individuals to report hazardous consumption.

**TABLE 2:** Odds ratios (OR) for hazardous drinking with 95% confidence intervals (CI), by migration characteristics.

		All		Women		Men	
		OR (95% CI)	OR (95% CI)	OR (95% CI)	OR (95% CI)	OR (95% CI)	OR (95% CI)
		Model 1	Model 2	Model 1	Model 2	Model 1	Model 2
Country/region of birth							
Sweden		Ref	Ref	Ref	Ref	Ref	Ref
Other Nordic countries		**1.43 (1.16–1.75)**	**1.45 (1.18–1.77)**	**1.73 (1.24–2.43)**	**1.72 (1.24–2.39)**	1.23 (0.98–1.55)	**1.27 (1.00–1.60)**
European countries		**0.54 (0.43–0.68)**	**0.55 (0.44–0.69)**	**0.41 (0.31–0.55)**	**0.42 (0.32–0.56)**	**0.63 (0.47–0.86)**	**0.64 (0.48–0.87)**
Non‐European countries		**0.25 (0.20–0.31)**	**0.25 (0.20–0.31)**	**0.24 (0.17–0.34)**	**0.24 (0.18–0.34)**	**0.26 (0.19–0.34)**	**0.26 (0.19–0.34)**
Duration of residence							
Swedish‐born		Ref	Ref	Ref	Ref	Ref	Ref
All migrants	≥20 years	**0.73 (0.61–0.88)**	**0.72 (0.60–0.86)**	0.81 (0.63–1.05)	0.80 (0.62–1.03)	**0.69 (0.54–0.88)**	**0.67 (0.53–0.85)**
All migrants	10–19 years	**0.41 (0.32–0.53)**	**0.41 (0.32–0.52)**	**0.47 (0.34–0.64)**	**0.46 (0.34–0.63)**	**0.38 (0.27–0.53)**	**0.38 (0.27–0.53)**
All migrants	<10 years	**0.24 (0.19–0.31)**	**0.26 (0.20–0.33)**	**0.13 (0.09–0.19)**	**0.13 (0.09–0.20)**	**0.32 (0.23–0.43)**	**0.34 (0.25–0.47)**
Age at migration							
Swedish‐born		Ref	Ref	Ref	Ref	Ref	Ref
All migrants	<7 years	0.80 (0.58–1.09)	0.79 (0.58–1.07)	1.05 (0.68–1.63)	1.03 (0.67–1.59)	**0.62 (0.41–0.92)**	**0.62 (0.42–0.91)**
All migrants	7–17 years	**0.45 (0.32–0.63)**	**0.45 (0.32–0.63)**	**0.58 (0.40–0.84)**	**0.58 (0.40–0.83)**	**0.38 (0.23–0.64)**	**0.39 (0.23–0.65)**
All migrants	18–30 years	**0.42 (0.34–0.53)**	**0.43 (0.35–0.53)**	**0.32 (0.24–0.43)**	**0.33 (0.25–0.44)**	**0.50 (0.37–0.67)**	**0.50 (0.38–0.66)**
All migrants	>30 years	**0.38 (0.31–0.48)**	**0.40 (0.32–0.50)**	**0.25 (0.17–0.38)**	**0.26 (0.17–0.39)**	**0.45 (0.35–0.60)**	**0.47 (0.36–0.62)**

*Note*: Separate regression analyses were performed for country /region of birth, duration of residence and age at migration, respectively. Model 1 adjusted for sex (except for sex stratified analyses), age at survey and survey year. Model 2 adjusted additionally for education, income and GHQ12. Bold estimates denote those which are statistically significant (*p* < 0.05).

A gradient in the odds of hazardous drinking in the migrant population, relative to the Swedish‐born population, was observed by duration of residence; ORs were 0.26 (95% CI 0.20–0.33) for migrants with a residence of less than 10 years, 0.41 (95% CI 0.32–0.52) for a residency of 10–19 years and 0.72 (95% CI 0.60–0.86) for those who had lived at least 20 years in Sweden. Differences by duration of residence were statistically significant (*p* < 0.001 for the difference between ≥20 years and other categories, and *p* = 0.007 for the difference between <10 and 10–19 years).

Age at migration to Sweden was also associated with odds of hazardous drinking. Individuals who migrated when they were 7 years or older were less likely to report hazardous drinking compared to Swedish‐born individuals (ORs ranging from 0.40 to 0.45). For individuals who came to Sweden as young children (i.e., before age seven), there was no statistically significant difference in odds relative to the Swedish‐born (OR 0.79, 95% CI 0.58–1.07). The difference in hazardous drinking between individuals who migrated before and after the age of seven was statistically significant (*p* < 0.001). The gradient in the odds of hazardous drinking in the migrant population by duration of residence was seen among individuals who arrived during childhood as well as in adulthood (Table S3).

Differences between migrant men and women in relation to hazardous drinking by duration of residence and age at migration were also observed. Among migrants with a short residence (<10 years in Sweden), there was a statistically significant interaction with gender (*p* = 0.001) with an OR of hazardous drinking of 0.13 (95% CI 0.09–0.20) among women and 0.34 (95% CI 0.25–0.47) among men, relative to Swedish‐born women and men, respectively. Among migrants with a longer residence, there was no statistically significant interaction with gender. Relative to their Swedish‐born counterparts, women who migrated at pre‐school ages showed similar OR for hazardous drinking (1.03, 95% CI 0.67–1.59), whereas men who migrated at the same ages had a significantly lower OR (0.62, 95% CI 0.42–0.91, *p* = 0.045 for interaction by gender). In contrast, among individuals who migrated in adulthood, lower ORs relative to the Swedish‐born population were seen among women compared to men (*p* = 0.026 [age at migration 18–30 years]; *p* = 0.004 [age at migration >30 years]).

ORs presented in Table 3 also demonstrate increased odds for Nordic migrants, relative to the Swedish‐born, however, with no distinct patterns by duration of residence or age at migration (results from post‐estimation tests presented in Table S2). For European and non‐European migrants, lower odds for hazardous drinking were observed, with the exception of non‐significant odds for European migrants with the longest duration of residence, which did not differ from the Swedish‐born (OR 0.79, 95% CI 0.56–1.12). Post‐estimation tests revealed that non‐European and European migrants with <10 years of residence had significantly lower odds relative to their counterparts with 20+ years of residence; gender‐stratified analyses showed that this pattern was particularly evident for women (*p*‐values from post‐estimation tests <0.05, see Table S2).

**TABLE 3:** Odd ratios (OR) for hazardous drinking with 95% confidence intervals (CI) in relation to duration of residence and age at migration, by region of origin.

		All migrants	Women	Men
		OR (95% CI)	OR (95% CI)	OR (95% CI)
Region of origin	Duration			
Sweden		Ref	Ref	Ref
Other Nordic	≥20 years	**1.47 (1.15–1.88)**	**1.77 (1.17–2.67)**	1.29 (1.00–1.66)
	10–19 years	1.55 (0.94–2.56)	**2.01 (1.11–3.65)**	1.24 (0.57–2.67)
	<10 years	1.31 (0.85–2.03)	1.37 (0.79–2.37)	1.25 (0.64–2.41)
European	≥20 years	0.79 (0.56–1.12)	**0.58 (0.38–0.88)**	0.93 (0.59–1.47)
	10–19 years	**0.40 (0.29–0.55)**	**0.52 (0.33–0.80)**	**0.32 (0.20–0.51)**
	<10 years	**0.42 (0.29–0.59)**	**0.19 (0.10–0.35)**	**0.62 (0.41–0.96)**
Non‐European	≥20 years	**0.38 (0.29–0.51)**	**0.53 (0.35–0.80)**	**0.31 (0.21–0.45)**
	10–19 years	**0.31 (0.20–0.48)**	**0.28 (0.15–0.50)**	**0.34 (0.19–0.60)**
	<10 years	**0.11 (0.07–0.18)**	**0.01 (0.004–0.05)**	**0.17 (0.10–0.29)**
Region of origin	Age at migration			
Sweden		Ref	Ref	Ref
Other Nordic	<7 years	**1.90 (1.03–3.51)**	2.50 (0.87–7.22)	1.54 (0.90–2.62)
	7–17 years	1.25 (0.87–1.79)	**1.71 (1.06–2.74)**	0.88 (0.51–1.52)
	≥18 years	**1.35 (1.10–1.67)**	**1.48 (1.07–2.03)**	1.27 (0.96–1.68)
European	<7 years	0.84 (0.54–1.31)	0.79 (0.44–1.44)	0.88 (0.47–1.65)
	7–17 years	0.61 (0.35–1.08)	0.58 (0.33–1.01)	0.64 (0.28–1.46)
	≥18 years	**0.48 (0.36–0.63)**	**0.30 (0.20–0.44)**	**0.61 (0.43–0.86)**
Non‐European	<7 years	**0.47 (0.30–0.72)**	0.81 (0.50–1.32)	**0.24 (0.12–0.48)**
	7–17 years	**0.21 (0.12–0.38)**	**0.32 (0.15–0.71)**	**0.16 (0.07–0.37)**
	≥18 years	**0.21 (0.16–0.29)**	**0.08 (0.04–0.15)**	**0.29 (0.21–0.40)**

*Note*: Separate regression analyses were performed for duration of residence and age at migration, respectively. Adjusted for sex (except for sex stratified analyses), age at survey and survey year, education, income, and psychological distress. Bold estimates denote those which are statistically significant (*p* < 0.05).

Gender differences within each origin group were only evident among European and non‐European migrants with less than 10 years of residence, whereby women were less likely than their male counterparts to report hazardous consumption (*p* = 0.004 for interaction by gender among European and *p* = 0.001 among non‐European migrants). Differences between men and women who had lived longer in Sweden were statistically non‐significant.

Among European and non‐European migrants, risks of hazardous drinking tended to be more similar to the Swedish‐born reference group with younger age at migration. Accordingly, the lowest odds were observed among European migrants who came to Sweden as adults (OR 0.48, 95% CI 0.36–0.63) and non‐European migrants who migrated during school age (OR 0.21, 95% CI 0.12–0.38) or as adults (OR 0.21, 95% CI 0.16–0.29); these estimates were statistically significantly different from individuals migrating before age seven (*p* < 0.05; Table S2). The gender stratified results further showed that this was primarily observed among non‐European migrant women, for whom decreased odds for hazardous drinking were seen among those who migrated at older ages but not for those who migrated before age 7.

## 4 DISCUSSION

The migrant population in Sweden displayed diverse patterns of hazardous alcohol consumption, which differed by origin and were clearly associated with duration of residence and age at migration. Overall patterns suggested hazardous consumption levels that were increasingly similar to the Swedish‐born population with longer residence and younger age of migration, albeit with different magnitudes by region of origin. Taken together, these findings broadly point to the important role of the settlement context in influencing alcohol consumption behaviours among migrants.

Relative to the Swedish‐born population, Nordic migrants reported increased risks of hazardous drinking, whereas a decreased likelihood of hazardous drinking was seen among European and non‐European migrants. These findings are comparable with reported levels of hazardous drinking from national Swedish surveys and align with previous research [10, 11, 12, 31]. The likelihoods of hazardous consumption among Nordic migrants were similar to or greater than among the Swedish‐born across all categories of duration of residence and age of migration; these findings likely reflect, at least in part, pre‐migration consumption patterns, which may be similar to or even higher than those of the Swedish‐born population [10]. However, for European and non‐European migrants, our findings point to convergence patterns in hazardous alcohol consumption with longer residence, similar to findings from other high‐income country contexts [2]. Relatedly, the patterns of association for age of migration correlated with those observed for duration of residence. Both European and non‐European migrants showed likelihoods of hazardous consumption that were below those of the Swedish‐born, but which increased with longer residence. This was most evident among European migrants, who showed convergence in hazardous consumption with 20+ years of residence in Sweden. European and non‐European migrants showed a gradient in the likelihood of hazardous consumption by age of arrival, with those who migrated in pre‐school ages showing consumption patterns that were the most similar to those of the Swedish‐born.

Compared to most other non‐Nordic country contexts, the Swedish alcohol policy approach is more restrictive, aimed at maintaining a low overall consumption in the general population. Key elements include a state alcohol retail monopoly, limitations on the physical availability of alcohol, taxation and price control, and stringent restrictions on marketing [15, 32]. Alcohol consumption levels in the general Swedish population have historically been low, but Swedish drinking culture has also been characterised as intoxication‐oriented, with infrequent heavy drinking and high rates of intoxication [33]. However, despite Sweden's restrictive policy context and overall low population levels of alcohol consumption, our findings demonstrate that certain migrant groups over time adopt levels of hazardous drinking that are more similar to the majority population. This pattern towards convergence aligns with similar findings from country contexts with liberal alcohol policies, such as North America or the United Kingdom [2]. As this pattern is observed in various contexts, irrespective of host country alcohol policy, the findings highlight the need for additional measures, including public health strategies, to reduce hazardous alcohol consumption.

In the literature, post‐migration changes in health behaviours are often explained by acculturation processes that impact attitudes and behaviours, whereby longer exposure to ‘mainstream’ society and culture is thought to explain consumption patterns that become more similar to those of the native‐born population [4]. Yet acculturation based explanations tend to largely ignore the role of socio‐historical contexts of migration [34], as well as the reception context and social and structural determinants of health in settlement, which may have implications for the development of hazardous consumption behaviours. As such, the current study's findings suggesting convergence in hazardous consumption may also be interpreted through critical perspectives on migration and health, such as cumulative inequalities [35], cultural stress [36], weathering [37] and exhausted migrant theories [38]. These critical perspectives consider the ways in which stressful life experiences related to migration and integration processes—including adapting to life in a new society, family separation, and experiences of inequalities and marginalisation and exclusionary processes such as social isolation, structural racism and discrimination—may negatively impact health or increase the risk of hazardous alcohol use as a coping mechanism. Yet, in addition to the role of duration of residence, our findings also highlight the importance of the life‐course stage during which the individual migrates for health behaviours. Individuals who migrate earlier and grow up in the settlement country may find it easier to adapt and integrate into the new society [39, 40], but may also be more likely to adopt health‐related norms and behaviours that are more similar to those of the native‐born population [41]. This may be particularly relevant for children and adolescents who migrate before critical developmental periods when health behaviours are most likely to be influenced by mainstream cultural norms or peer influences [42].

Finally, it should also be noted that patterns of hazardous consumption in some migrant groups diverged by gender. The risk of hazardous drinking among non‐European migrant women who migrated during early childhood was similar to that of Swedish‐born women, whereas non‐European men had a lower risk relative to Swedish‐born men, regardless of their age at migration. Among migrants from other European countries, gender differences (with women showing lower odds compared to men) were evident only among those who migrated in adulthood and those with shorter durations of residence. Gender differences in alcohol consumption patterns are consistent findings in the literature, although the size of the differences varies across societies and over time. The smallest gender differences are generally seen in Europe, particularly in the Nordic countries [43, 44]. Additionally, research has indicated increasing levels of heavy episodic drinking among women, further diminishing the gender gap [45, 46]. It has been suggested that these converging patterns may be partly explained by societal factors, such as women's position in society, including increased participation in the labour market and changing economic conditions [45, 46]. Our findings, which indicate that changes over time were more prominent among women, may to some extent reflect these general patterns and changes in attitudes towards drinking, gender roles and norms, that is, what is considered socially acceptable in terms of alcohol consumption. The importance of reflecting on gender differences in migration‐related research has gained recognition [26], yet few studies explore intersections between migration background characteristics, gender and consumption behaviours. Our findings suggest that the timing of migration in the life course may have different implications for hazardous drinking between men and women, but further empirical research is needed.

Our study has several strengths, including the use of register‐linked survey data and a large migrant study population that made analyses of intersections between migration background characteristics and gender possible. Another strength was the use of a validated screening tool to capture hazardous drinking. Although our survey data was limited to one geographic region, the proportions of migrants sampled was similar to that of the entire country, suggesting that our findings are generalisable to the broader Swedish context. The survey data was collected in 2011 and 2015, and as such generalizability of the findings to the current migrant population in Sweden should also be considered. Even though migration policies have become more restrictive and migration has declined since 2016, which may affect the composition of newly arrived migrants, the overall population remains diverse by origin, duration and age of arrival. Furthermore, although Sweden's alcohol policy has undergone some changes during the past decades (e.g., an expanded e‐commerce), the fundamental principles with restricted availability and high taxation and prices have remained largely the same.

The main limitations of the current study included the lower response rate among migrants, which is not uncommon in survey‐based research [47], and the cross‐sectional nature of the data material, which hampered the evaluation of individual behavioural changes over time. Another potential limitation is that available data included rather broad categories of migrant origin. However, given our ambition to also study combinations of migration background factors by gender, use of more detailed information by origin would have resulted in a lack of sufficient statistical power. Data on parental country of birth was not available, and the lack of information about individuals born in Sweden with at least one foreign‐born parent is another limitation. Furthermore, as we cannot distinguish effects by duration of residence from potential differences over time in the migrant population, that is, depending on their year of arrival, we cannot explore potential cohort effects. However, the effect of duration of residence on various outcomes is well established in the literature and our results mirror patterns seen in various settings and migrant cohorts [2].

In conclusion, our study found that migrants in Sweden had lower odds of hazardous drinking than the Swedish‐born population, particularly those with shorter durations of residence and those who migrated as adults. However, migrants with longer residency and younger ages at arrival had risks of hazardous drinking comparable to those of Swedish‐born individuals. Increased understanding of how consumption patterns and hazardous drinking vary across migrant groups, evolve over time, and differ between policy contexts is essential for improving public health strategies and developing effective prevention measures to reduce hazardous drinking and minimise related health and social harms.

## AUTHOR CONTRIBUTIONS

Each author certifies that their contribution to this work meets the standards of the International Committee of Medical Journal Editors.

## CONFLICT OF INTEREST STATEMENT

The authors have no conflicts of interest to declare.

## Supporting information


**Data S1.** Supporting information.

## Data Availability

The authors do not have permission to share data.
