# Supplementary material for: Hazardous drinking by age at migration and duration of residence among migrants in Sweden
Source: Drug Alcohol Rev. 2024 Nov 25;44(2):480–90. doi: 10.1111/dar.13982 (PMC11814355; doi:10.1111/dar.13982)
Supplement: Supplementary file 1 — Data S1. Supporting information. [file DAR-44-480-s001.docx]

**Table S1.** Distribution of AUDIT-C scores across region of origin

| **AUDIT-C score** | **Sweden** | **Other Nordic countries** | **European countries** | **Non-European countries** |
| --- | --- | --- | --- | --- |
| **0** | 13.2% | 17.1% | 25.9% | 41.1% |
| **1** | 15.3% | 16.7% | 21.1% | 20.4% |
| **2** | 19.0% | 17.55 | 17.6% | 14.2% |
| **3** | 17.8% | 16.1% | 12.6% | 8.5% |
| **4** | 15.5% | 13.4% | 10.6% | 6.7% |
| **5** | 8.2% | 7.9% | 5.8% | 3.8% |
| **6** | 5.0% | 5.0% | 3.0% | 2.6% |
| **7** | 2.9% | 2.5% | 1.9% | 1.2% |
| **8** | 1.9% | 2.3% | 1.0% | 0.6% |
| **9** | 0.8% | 0.9% | 0.4% | 0.5% |
| **10** | 0.4% | 0.5% | 0.2% | 0.3% |
| **11** | 0.1% | 0.2% | 0 | 0.04% |
| **12** | 0.05% | 0.08% | 0 | 0 |

AUDIT-C, Alcohol Use Disorders Identification Test.

**Table S2.** Odd Ratios (OR) for hazardous drinking with 95% confidence intervals (CI), in relation to duration of residence and age at migration, by region of origin

|  |  | **All migrants** | **Post-estimation tests** | **Women** | **Post-estimation tests** | **Men** | **Post-estimation tests** |
| --- | --- | --- | --- | --- | --- | --- | --- |
|  |  | OR (95% CI) | *p*-value* | OR (95% CI) | *p*-value* | OR (95% CI) | *p*-value* |
| **Region of origin** | **Duration** |  |  |  |  |  |  |
| Sweden |  | Ref |  | Ref |  | Ref |  |
| Other Nordic | ≥20 years | **1.47 (1.15-1.88)** |  | **1.77 (1.17-2.67)** |  | 1.29 (1.00-1.66) |  |
|  | 10-19 years | 1.55 (0.94-2.56) | 0.8565 | **2.01 (1.11-3.65)** | 0.7207 | 1.24 (0.57-2.67) | 0.9229 |
|  | <10 years | 1.31 (0.85-2.03) | 0.6149/0.6434 | 1.37 (0.79-2.37) | 0.3476/0.4667 | 1.25 (0.64-2.41) | 0.9861/0.9314 |
| European | ≥20 years | 0.79 (0.56-1.12) |  | **0.58 (0.38-0.88)** |  | 0.93 (0.59-1.47) |  |
|  | 10-19 years | **0.40 (0.29-0.55)** | 0.0044 | **0.52 (0.33-0.80)** | 0.7184 | **0.32 (0.20-0.51)** | 0.0011 |
|  | <10 years | **0.42 (0.29-0.59)** | 0.8394/0.0107 | **0.19 (0.10-0.35)** | 0.0103/0.0039 | **0.62 (0.41-0.96)** | 0.0348/0.2017 |
| Non-European | ≥20 years | **0.38 (0.29-0.51)** |  | **0.53 (0.35-0.80)** |  | **0.31 (0.21-0.45)** |  |
|  | 10-19 years | **0.31 (0.20-0.48)** | 0.4315 | **0.28 (0.15-0.50)** | 0.0767 | **0.34 (0.19-0.60)** | 0.7926 |
|  | <10 years | **0.11 (0.07-0.18)** | 0.0021/0.0000 | **0.01 (0.004-0.05)** | 0.0000/0.0000 | **0.17 (0.10-0.29)** | 0.0876/0.074 |
|  |  |  |  |  |  |  |  |
| **Region of origin** | **Age at migration** |  |  |  |  |  |  |
| Sweden |  | Ref |  | Ref |  | Ref |  |
| Other Nordic | <7 years | **1.90 (1.03-3.51)** |  | 2.50 (0.87-7.22) |  | 1.54 (0.90-2.62) |  |
|  | 7-17 years | 1.25 (0.87-1.79) | 0.250 | **1.71 (1.06-2.74)** | 0.5187 | 0.88 (0.51-1.52) | 0.1521 |
|  | ≥18 years | **1.35 (1.10-1.67)** | 0.7080/0.3095 | **1.48 (1.07-2.03)** | 0.6094/0.3519 | 1.27 (0.96-1.68) | 0.2377/0.5367 |
| European | <7 years | 0.84 (0.54-1.31) |  | 0.79 (0.44-1.44) |  | 0.88 (0.47-1.65) |  |
|  | 7-17 years | 0.61 (0.35-1.08) | 0.3766 | 0.58 (0.33-1.01) | 0.4448 | 0.64 (0.28-1.46) | 0.5393 |
|  | ≥18 years | **0.48 (0.36-0.63)** | 0.4445/0.0321 | **0.30 (0.20-0.44)** | 0.0566/0.0070 | **0.61 (0.43-0.86)** | 0.9172/0.3096 |
| Non-European | <7 years | **0.47 (0.30-0.72)** |  | 0.81 (0.50-1.32) |  | **0.24 (0.12-0.48)** |  |
|  | 7-17 years | **0.21 (0.12-0.38)** | 0.0316 | **0.32 (0.15-0.71)** | 0.0494 | **0.16 (0.07-0.37)** | 0.4603 |
|  | ≥18 years | **0.21 (0.16-0.29)** | 0.9985/0.0035 | **0.08 (0.04-0.15)** | 0.0061/0.0000 | **0.29 (0.21-0.40)** | 0.1944/0.6464 |

* For the 10-19 years category the *p*-value is for comparison with the ≥20 years category. For the <10 years category the p-values are for comparisons with the 10-19 years category/the ≥20 years category.

|  | **All migrants** | | **<17 years at arrival** | | **>=18 years at arrival** | |
| --- | --- | --- | --- | --- | --- | --- |
|  |  | p-value from post-estimation test* | OR (95% CI) | *p*-value from post-estimation test* | OR (95% CI) | *p*-value from post-estimation test* |
| **Duration of residence** |  |  |  |  |  |  |
| Swedish-born | Ref |  | Ref |  | Ref |  |
| ≥20 years | **0.55 (0.43-0.69)** |  | **0.66 (0.47-0.92)** |  | **0.46 (0.33-0.66)** |  |
| 10-19 years | **0.35 (0.27-0.46)** | 0.0157 | **0.40 (0.26-0.60)** | 0.0597 | **0.32 (0.22-0.46)** | 0.1392 |
| <10 years | **0.21 (0.16-0.28)** | 0.0097/0.0000 | **0.13 (0.06-0.28)** | 0.0121/0.0001 | **0.23 (0.17-0.31)** | 0.1646/0.0026 |

**Table S3.** Odd ratios (OR) for hazardous drinking with 95% confidence intervals (CI) by duration of residence, among European and non-European migrants

* For the 10-19 years category the p-value is for comparison with the ≥20 years category. For the <10 years category the *p*-values are for comparisons with the 10-19 years category and the ≥20 years category, respectively.
